# Supplementary material for: Ultrapotent bispecific antibodies neutralize emerging SARS-CoV-2 variants
Source: bioRxiv. 2021 Apr 1:2021.04.01.437942. Preprint. [Version 1] doi: 10.1101/2021.04.01.437942 (PMC8020967; doi:10.1101/2021.04.01.437942)
Supplement: Supplement 3 [file media-3.pdf]

Supplementary Table 2. X-ray data collection and refinement statistics

**Data collection**	CV503 + RBD + COVA1-16
Beamline	SSRL12-1
Wavelength (Å)	0.97946
Space group	P 1 2_1 1
Unit cell parameters	
a, b, c (Å)	172.2, 122.7, 175.5
α, β, γ (°)	90, 118.2, 90
Resolution (Å) [a]	50.0-3.40 (3.48-3.40)
Unique reflections [a]	87,443 (8,562)
Redundancy [a]	3.4 (3.5)
Completeness (%) [a]	98.8 (99.8)
$<I/\sigma_I>$ [a]	10.2 (1.0)
R_{sym}[b] (%) [a]	14.6 (>100)
R_{pim}[b] (%) [a]	4.3 (64.9)
$CC_{1/2}$[c] (%) [a]	99.8 (53.6)
**Refinement statistics**	
Resolution (Å)	40.6-3.40
Reflections (work)	87,416
Reflections (test)	2,000
R_{cryst}[d] / R_{free}[e] (%)	20.0/23.5
No. of atoms	24,307
RBD	4,671
CV503 Fab	9,602
COVA1-16 Fab	10,034
Average *B*-values (Å^2)	131
RBD	139
CV503 Fab	131
COVA1-16 Fab	128
Wilson *B*-value (Å^2)	128
**RMSD from ideal geometry**	
Bond length (Å)	0.002
Bond angle (°)	0.60
**Ramachandran statistics (%)**	
Favored	95.8
Outliers	0.28
**PDB code**	pending

[a] Numbers in parentheses refer to the highest resolution shell.

[b] $R_{sym} = \Sigma_{hkl} \Sigma_i | I_{hkl,i} - <I_{hkl}> | / \Sigma_{hkl} \Sigma_i I_{hkl,i}$ and $R_{pim} = \Sigma_{hkl} (1/(n-1))^{1/2} \Sigma_i | I_{hkl,i} - <I_{hkl}> | / \Sigma_{hkl} \Sigma_i I_{hkl,i}$, where $I_{hkl,i}$ is the scaled intensity of the ith measurement of reflection h, k, l, $<I_{hkl}>$ is the average intensity for that reflection, and *n* is the redundancy.

[c] $CC_{1/2}$ = Pearson correlation coefficient between two random half datasets.

[d] $R_{cryst} = \Sigma_{hkl} | F_o - F_c | / \Sigma_{hkl} | F_o | \times 100$, where F_o and F_c are the observed and calculated structure factors, respectively.

[e] R_{free} was calculated as for R_{cryst}, but on a test set comprising 5% of the data excluded from refinement.
